# Assessing Grapevine Biophysical Parameters From Unmanned Aerial Vehicles Hyperspectral Imagery

**DOI:** 10.3389/fpls.2022.898722

**Published:** 2022-06-02

**Authors:** Alessandro Matese, Salvatore Filippo Di Gennaro, Giorgia Orlandi, Matteo Gatti, Stefano Poni

**Affiliations:** ^1^Institute of BioEconomy, National Research Council (CNR-IBE), Firenze, Italy; ^2^Department of Sustainable Crop Production (DI.PRO.VE.S.), Università Cattolica del Sacro Cuore, Piacenza, Italy

**Keywords:** unmanned aerial vehicles (UAV), precision viticulture, hyperspectral sensing, vegetation indices, image segmentation

## Abstract

Over the last 50 years, many approaches for extracting plant key parameters from remotely sensed data have been developed, especially in the last decade with the spread of unmanned aerial vehicles (UAVs) in agriculture. Multispectral sensors are very useful for the elaboration of common vegetation indices (VIs), however, the spectral accuracy and range may not be enough. In this scenario, hyperspectral (HS) technologies are gaining particular attention thanks to the highest spectral resolution, which allows deep characterization of vegetative/soil response. Literature presents few papers encompassing UAV-based HS applications in vineyard, a challenging conditions respect to other crops due to high presence of bare soil, grass cover, shadows and high heterogeneity canopy structure with different leaf inclination. The purpose of this paper is to present the first contribution combining traditional and multivariate HS data elaboration techniques, supported by strong ground truthing of vine ecophysiological, vegetative and productive variables. Firstly the research describes the UAV image acquisition and processing workflow to generate a 50 bands HS orthomosaic of a study vineyard. Subsequently, the spectral data extracted from 60 sample vines were elaborated both investigating the relationship between traditional narrowband VIs and grapevine traits. Then, multivariate calibration models were built using a double approach based on Partial Least Square (PLS) regression and interval-PLS (iPLS), to evaluate the correlation performance between the biophysical parameters and HS imagery using the whole spectral range and a selection of more relevant bands applying a variable selection algorithm, respectively. All techniques (VIs, PLS and iPLS) provided satisfactory correlation performances for the ecophysiological (*R*^2^ = 0.65), productive (*R*^2^ = 0.48), and qualitative (*R*^2^ = 0.63) grape parameters. The novelty of this work is represented by the first assessment of a UAV HS dataset with the expression of the entire vine ecosystem, from the physiological and vegetative state to grapes production and quality, using narrowband VIs and multivariate PLS regressions. A correct non-destructive estimation of key parameters in vineyard, above all physiological parameters which must be measured in a short time as they are extremely influenced by the variability of environmental conditions during the day, represents a powerful tool to support the winegrower in vineyard management.

## Introduction

**FIGURE 1:**
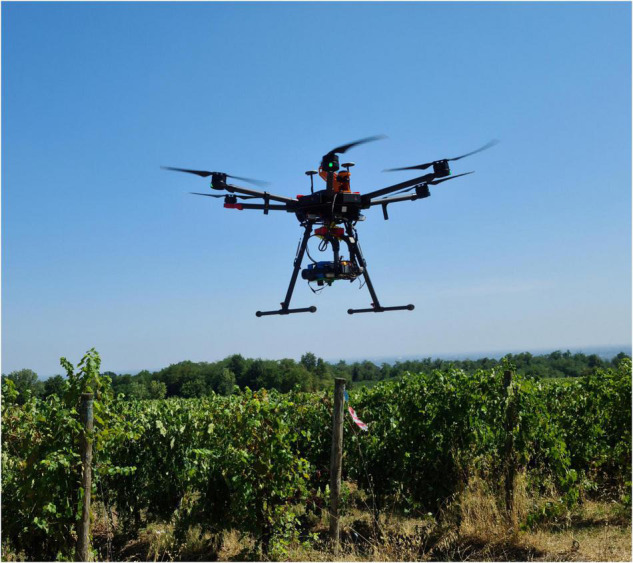
Unmanned Aerial Vehicle used in the study equipped with hyperspectral (HS) imaging sensor.

To measure the dynamic response of plants to changing environmental conditions, quantitative vegetation variable extraction is essential. The spatiotemporally explicit retrieval of plant biophysical characteristics is possible using Earth observation sensors in the optical domain. Satellite remote sensing has been widely employed in agriculture during the last few decades (Immitzer et al., 2012; Pastonchi et al., 2020; Squeri et al., 2021). Unmanned aerial vehicles (UAVs) have recently attracted a lot of attention because of their increased mission schedule flexibility, acquiring data with higher spatial resolution in a precision viticulture context (Adão et al., 2017). Over the last 50 years, many approaches for extracting biophysical and biochemical parameters from remotely sensed data have been developed. In this context, UAV based HS sensors (Figure 1) are gaining particular attention due to their well-known ability to provide deep spectral characterization of vegetation and soil targets. HS imagery has been applied to quantify leaf area index (Haboudanea et al., 2004; Delegido et al., 2013), plant biomass (Cho et al., 2007; Fu et al., 2014), pigment contents (Yi et al., 2014), plant nitrogen content (Ryu et al., 2011; Inoue et al., 2012), and leaf nitrogen and phosphorus concentrations (Ramoelo et al., 2013; Zhang et al., 2013), soil moisture content (Ge et al., 2021), as well as plant water status and transpiration (Wang and Jin, 2015; Marshall et al., 2016).

Traditionally HS imaging sensors have been manufactured with a push-broom line scanning approach (Suomalainen et al., 2014). Recently, hyperspectral sensing technologies that acquire two-dimensional frame format have entered the market (Aasen et al., 2018). Senop HSC-2 HS camera (Senop Optronics, Finland) is characterized by a global shutter snapshot sensor, a tuneable Fabry-Pérot interferometer, able to record data in the VNIR (Visible and Near Infrared) spectral range 500–900 nm; Cubert UHD 185-Firefly (Cubert GmbH, Germany) and the IMEC SM5 × 5 (IMEC, Belgium) sensors provide registered bands frames. Photogrammetric experiments using unmanned airborne vehicles (UAVs) may also benefit from scaled-down hyperspectral 2D cameras, making for a more cost-effective mapping process. HS imagery captured by UAVs has mostly been used for agricultural and environmental surveillance (Aasen et al., 2018; Oliveira et al., 2019). As a result, the collection and interpretation of UAV-derived data has become easier, faster, and more accurate.

When operating on a UAV, the Senop camera acquires hypercubes from various spectral ranges and bands, and emits non-registered bands. To prevent band misalignment, co-registration is required. The capacity of various 2D shifts in band registration of time-consecutive camera images was tested by Tommaselli et al. (2015). Honkavaara et al. (2013) found that in flat agricultural scenarios, band registration of such images with feature-based matching and 2D image transformation provided successful registration. MEPHySTo was introduced by Jakob et al. (2017) as a toolbox for pre-processing UAV HS data, consisting of a pre-processing chain optimized for difficult geometric and radiometric correction. It also includes automated mosaicking and georeferencing algorithms that allow for quick and simple surveying of remote areas where obtaining ground control points (GCP) would be difficult or time-consuming.

The retrieval of biophysical parameters from HS data could be evaluated using parametric regression with discrete band approaches (vegetation indices—VIs) or quasi-continuous spectral bands, or linear/non-linear non-parametric regression with linear (partial least square regression—PLSR) or non-linear non-parametric regression (random forest, support vector machine—SVM, gaussian process regression—GPR) (Matese and Di Gennaro, 2021). Many VIs depend on a combination of near-infrared (NIR) and red reflectance, such as the NIR-to-red ratio. While most structural indices were built using broad-band systems, narrow-band (<10 nm) equivalents can be measured through HS imagery. On the other hand, several biochemical/physiological indices are simply hyperspectral requiring small bands (=10 nm) and non-sample band centers that are not considered by broad-band systems. Several HS-derived VIs (HVIs) based on narrow bands have been proposed for quantifying biophysical parameters since the advent of HS remote sensing, offering additional information and significant advantages over large bands (Thenkabail et al., 2000). Transformed spectrum formats, such as transmittance and derivative spectra, have also been shown to be useful in generating more broadly available VIs for deriving biophysical and biochemical parameters. Derivative techniques, for example, have the advantage of minimizing additive constants and linear functions, allowing for remote sensing of crop parameters (Imanishi et al., 2004).

Traditional methods have been commonly used in post-processing for their ease of manipulation, such as those focused on VIs, stepwise multiple linear regression, partial least-squares regression, and so on (Dorigo et al., 2007). Broadband VIs date back to the 1970s and are primarily focused on multispectral remotely sensed data. The ratio vegetation index (RVI) (Pearson and Miller, 1972), normalized difference vegetation index (NDVI) (Rouse et al., 1974) and soil-adjusted vegetation index (SAVI) (Huete, 1988) are all common broadband VIs that were designed to eliminate the effects of environment and soil interferences. Many hyperspectral VIs (HVIs) based on narrow bands and very high spectral resolution have also been described since the advent of hyperspectral remote sensing. HVIs have long been used to estimate biophysical and biochemical attributes (Rodríguez-Pérez et al., 2007; Marshall et al., 2016). Despite the fact that some HVIs are closely copied or imitated from their broadband equivalents, it has been proposed that narrow bands may offer additional information and have important advantages over large bands in quantifying biophysical parameters (Thenkabail et al., 2000). Indeed, traditionally used VIs have many intrinsic shortcomings despite their ease of understanding and implementation (Baret and Guyot, 1991; Li and Wang, 2011). Such flaws can only be addressed by either increasing the dataset used to calculate VIs or enhancing the accuracy of that data. While the recently developed hybrid method significantly expanded the data volume and hence the likelihood of creating a more broadly usable VI, a variety of transformed spectra formats, such as transmittance and derivative spectra, have also proven to be effective in determining biophysical and biochemical parameters (Rady et al., 2014). Derivative methods, for example, have been shown to be feasible for estimating plant biophysical and biochemical parameters because they minimize additive constants and linear functions (Imanishi et al., 2004). For example, in plant condition detection, the red-edge location (REP), which is the wavelength of the maximum first derivative in the range of 690–750 nm, has been successfully used. As a result, a number of derivative hyperspectral indices (dHVIs) have been developed and are now being used to calculate biophysical and biochemical quantities (Demetriades-Shah et al., 1990; Imanishi et al., 2004; Wang and Jin, 2015). Demetriades-Shah et al. (1990) and Zarco-Tejada et al. (2003a,b) found that indices based on derivative spectra are more efficient than reflectance-based indices. However, the advantages of dHVIs over reflectance-based VIs, as well as the distinctions between derivatives of different orders, have yet to be thoroughly explored. The significant collinearity in spectral data must be considered when using statistical models for the retrieval of vegetative biophysical characteristics, and full spectrum techniques like PCA and PLS are extensively employed in chemometrics (Wold et al., 1987, 2001). These methods modify the spectral feature space so that the resultant (latent) factors account for the most variation in the feature space (PCA), or in the covariance with the target variables (PLS). State-of-the-art research presents only 7 papers encompassing UAV-based HS applications in a vineyard (Zarco-Tejada et al., 2012, 2013; Vanegas et al., 2018; Horstrand et al., 2019; Maimaitiyiming et al., 2020; Suarez et al., 2021; Di Gennaro et al., 2022), while more than 53 papers focused on HS applications in a vineyard without the use of UAVs. Di Gennaro et al. (2022) suggested a comparison in term of accuracy between broadband multispectral and narrowband HS data by means the calculation of some VIs on canopy and soil targets in vineyard, assessing in general higher spectral accuracy of HS camera respect to the ground truth provided by reference spectroradiometer (Di Gennaro et al., 2022). Suarez et al. (2021) investigated the links between grape quality parameters such as aroma components vs. image-based spectral indices and photosynthetic plant traits derived by physical model inversion methods. Maimaitiyiming et al. (2020) considered aerial hyperspectral and thermal images acquired by using a visible and near-infrared (VNIR, 400–1,000 nm) push-broom hyperspectral camera (Nano-Hyperspec VNIR model, Headwall Photonics, Fitchburg, MA, United States) installed in tandem with a thermal camera (FLIR Vue Pro R 640, FLIR Systems, Inc., Wilsonville, OR, United States) carried by a hexacopter (Matrice 600 Pro, DJI Technology Co., Ltd., Shenzhen, China). The authors proposed a canopy zone-weighting (CZW) method to estimate physiological indicators, such as stomatal conductance (gs) and steady-state fluorescence (Fs). Horstrand et al. (2019) used a solution based on a commercial DJI Matrice 600 and a Specim FX10 hyperspectral camera to adapt this latter device, mainly conceived for industrial applications, into a flying platform in which weight, power budget, and connectivity are paramount. Vanegas et al. (2018) used an S800 EVO Hexacopter (DJI Ltd., Shenzhen, China) combined with a Headwall Nano-Hyperspec (Headwall Photonics Inc., Bolton, MA, United States) for developing a predictive model aimed at detecting phylloxera infections. Zarco-Tejada et al. (2012, 2013) estimated leaf carotenoid content and water stress in vineyards by considering the same HS camera using narrowband indices. Other interesting studies focused on retrieving biophysical parameters in vineyards even not involving the use of UAVs are reported by Martin et al. (2007) and García-Estévez et al. (2017) who used hyperspectral imagery to map grape quality in “Tempranillo” vineyards, Haboudane et al. (2008) for crop chlorophyll content using derivatives spectral indices, while Pérez-Priego et al. (2015) investigated nutrient uptake. Although several authors focused on the evaluation of hyperspectral reflectance indices to detect grapevine water status (Rodríguez-Pérez et al., 2007; Serrano et al., 2012), only Pôças et al. (2017, 2020) used machine learning methodologies to obtain more detailed results.

Little work has been done about the benefits that might derive from characterizing efficiency parameters pertaining to the vineyard ecosystem from UAV platforms equipped with HS sensors. Moreover, the study has the ambition to move beyond traditional methodologies such as the use of VIs while testing and validating multivariate methods such as PLS, seeking for the significant bands in the characterization of the variables of interest. These objectives are crucial for the technological transfer to winegrowers, either for a better understanding of the vineyard characteristics and as valid tools to achieve winegrowers’ oenological objectives. In detail two main aims were pursued: (i) describe the image acquisition and processing workflow of HS data cubes developed in this work; (ii) test the performance of UAV equipped with an HS camera in grapevine ecophysiological, vegetative, productive and grape composition traits characterization, using narrowband HS-derived VIs and PLS models.

## Materials and Methods

### Experimental Site

**FIGURE 2:**
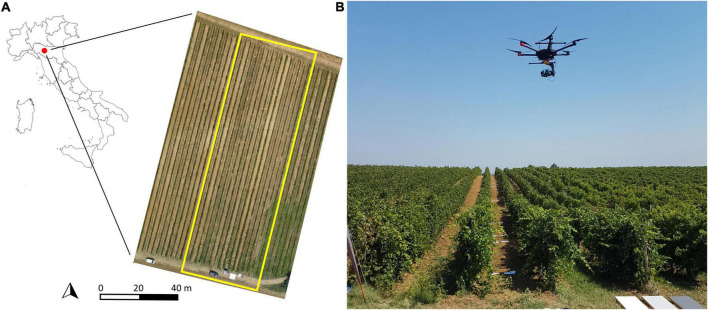
Location of the experimental vineyard **(A)** and the Unmanned Aerial System used in the study **(B)**.

The study was conducted in 2020 in a 15 rows plot placed within a rainfed Barbera vineyard established in 2003 at Tenuta Pernice (Castelnovo Val Tidone, Italy) (Figure 2A). Vines are spaced 2.4 × 1 m (between- and within-row, respectively), long-cane pruned and trained to a vertical-shoot-positioned trellis along NS oriented rows. During the season the canopy was trimmed twice on DOY (day of year) 162 and DOY 204, whilst vineyard management was performed according to organic farming protocols.

### System Description

A Senop HSC-2 HS camera mounted on a DJI Matrice Pro Hexacopter UAV platform (Figure 2B) was used for the flight on DOY 223 (10 August). The camera has a global shutter snapshot sensor that records data between the wavelengths of 500 and 900 nm. It has two partly reflecting surfaces that are parallel to each other. The length of the optical path provided between these reflecting surfaces (gap) determines the wavelengths that can be transmitted by the interferometer (Honkavaara et al., 2013). Various wavelengths can be obtained by adjusting this length. If the camera platform changes during the spectral band acquisition process, any spectral band in the same cube exposed to a particular air gap value has a different position and attitude (Honkavaara et al., 2013). The hyperspectral cube bands obtained with the camera can be modified based on individual applications and the camera’s spectral range and resolution. The image has a resolution of 1,024 × 1,024 pixels. The Senop camera has a beam splitting system and two CMOS sensors (without the Bayer filter) mounted (Oliveira et al., 2016a): the first is optimized to sense visible bands (500–636 nm), while the second is optimized to record both visible and NIR (650–900 nm). The flights were performed at a speed of 1.8 m/s at a height of 32 m above ground level (AGL) providing spectral images with a ground sampling distance (GSD) of approximately 2 cm/pixel. Front and side overlapping were 75 and 72%, respectively. The number and spectral sensitivities of the bands and integration time are the key parameters to be set. Time of integration was chosen as 1 ms in order to avoid image overexposure in relation to bright objects. The HSC-2 camera was set with 50 spectral bands (500–900) with a Full Width at Half Maximum (FWHM) of about 8 nm. Two types of reference were assessed; firstly, for reflectance conversion, five Senop targets with 2, 9, 25, 50, and 88% reflectance with sizes 50 × 50 cm. The targets are made of materials with nearly Lambertian reflectance properties and calibrated in laboratory conditions. Secondly, for geometric correction and georeferencing, white plastic targets with size 15 × 15 cm were used as ground control points (GCPs) and placed on the boundary of the test area as well as on the right side of 60 sample vines chosen for ground truth measurements, as described below.

### Ground Measurements

At full bloom (DOY 157) a pool of 60 vines was randomly identified within the 15-row plot and georeferenced by using a GPS. Per each sentinel vine, all clusters were picked, counted and weighed at harvest on DOY 260. In parallel, three representative basal-clusters per vine were collected and immediately taken to the laboratory for subsequent morphological and chemical characterization. Accordingly, from each sample, a 50-berry subsample was randomly collected and weighed to assess the mean berry weight. Grapes were then immediately frozen and stored at –18^°^C for subsequent determination of total anthocyanins and phenolics concentration (Iland, 1988). The remaining grapes were crushed for assessing total soluble solids (TSS) concentration, must pH and titratable acidity (TA). An aliquot of juice was diluted 1:4 with distilled water and used for quantifying the malic acid concentration as reported in Gatti et al. (2020). At onset of veraison (DOY 213), when full canopy growth was reached, pre-dawn (Ψpd) and mid-day (Ψmd) leaf water potential was determined by using a Scholander pressure chamber. Two leaves per plant were collected from a batch of 30 vines out of the 60 sentinel plants. After leaf fall, total nodes per vine on main and lateral dormant shoots was counted. Leaf area (LA) per vine was then calculated by multiplying the node number and corresponding mean values of leaf area for primary and lateral leaves as assessed at harvest (Gatti et al., 2021). In winter, during pruning operations performed on DOY 345, pruning weight for one-year-old canes was quantified by using a portable field-scale.

### Data Processing and Analysis

**FIGURE 3:**
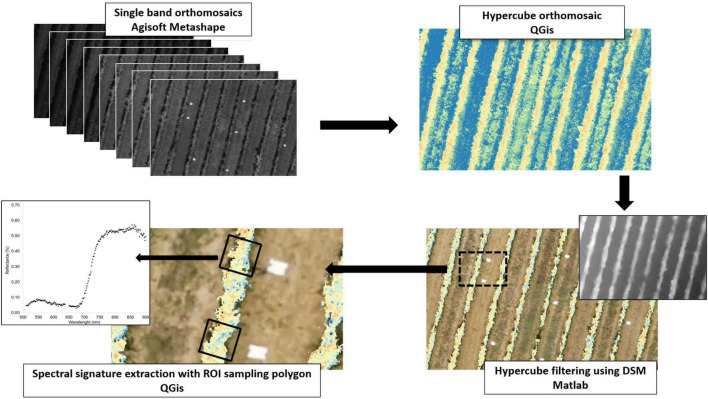
Hyperspectral images processing workflow.

The initial step was the conversion from DN to radiance, which was done with the use of factory calibration gains of the HS camera. Secondly, noise signals were removed from each image by means of the dark current measurement subtracted from radiance values. Finally, an empirical line method (ELM) was applied for the radiation to reflectance conversion (Matese et al., 2019), using five reference reflectance panels to perform radiometric correction for each band of the HS images. The next step was the HS orthomosaic generation, which is described in Figure 3. Agisoft Metashape software (AgiSoft LLC., St. Petersburg, Russia) was used for the reconstruction of each single band orthomosaic. After that a supervised procedure of georeferencing using GCPs was performed in QGIS software^1^ (2021. QGIS Geographic Information System. QGIS Association).^2^ In this work 215 hypercubes were acquired to monitor the whole study site. Once the full orthomosaic had been processed, Matlab software (MathWorks, Natick, Massachusetts, United States) was used to perform a segmentation procedure applying the DEM (Digital Elevation Model) method described in Cinat et al. (2019) and a further threshold filter was applied to avoid shaded leaves and soil. Finally, a supervised region-of-interest (ROI) procedure based on 0.8 × 0.8 m polygons was used for the HS data extraction from each sample vine, to perform the dataset post-processing in terms of retrieval of ground agronomic variables sampled using HS derived VIs.

Afterward, the following narrowband VIs (Table 1) were calculated using 50 bands hypercubes at very high spectral resolution (10 nm intervals) in the visible (VIS), Red Edge (RE), and near infrared (NIR) wavelengths for each polygon. Spectral pre-treatment was done using mean centering. The average of the VIs was calculated within each polygon.

**TABLE 1:** Narrowband vegetation indices calculated from HS dataset.

Vis	Full name	Spectral band group	Equation
NDVI 1	Normalized difference vegetation index	NIR, VIS	(R_850_-R_660_)/(R_850_ + R_660_)
NDVI 2	Normalized difference vegetation index	NIR, VIS	(R_835_-R_660_)/(R_835_ + R_660_)
NDVI 3	Normalized difference vegetation index	NIR, VIS	(R_850_-R_660_)/(R_850_ + R_660_)
GNDVI 1	Green NDVI	NIR, VIS	(R_850_-R_540_)/(R_850_ + R_540_)
GNDVI 2	Green NDVI	NIR, VIS	(R_780_-R_550_)/(R_780_ + R_550_)
SAVI	Soil-adjusted vegetation index	NIR, VIS	(1 + 0.5) × (R_802_-R_660_)/(R_802_ + R_660_ + 0.5)
RENDVI	Red edge normalized difference vegetation index	NIR, RE	(R_850_-R_680_)/(R_850_ + R_680_)
NDRE	Normalized difference nir/red edge index	NIR, RE	(R_770_-R_750_)/R_770_ + R_750_)
NRER	Nir-re-red normalized difference vegetation index	NIR, RE, VIS	(R_850_-R_695_)/(R_695_ + R_660_)
TCARI	Transformed chlorophyll absorption ratio	NIR, RE, VIS	3×[(R_695_-R_663_) –0.2(R_695_-R_540_) × (R_695_/R_663_)]
MTVI 1	Modified triangular vegetation index	NIR, RE, VIS	1.2× (1.2(R_800_-R_540_) –2.5(R_660_-R_540_)
MTVI 2	Modified triangular vegetation index	NIR, RE, VIS	1.2× (1.2(R_800_-R_550_) –2.5(R_670_-R_550_)
EVI	Enhanced vegetation index	NIR, RE, VIS	2.5× (R_850_-R_660_)/(R_850_ + 6×R_660_-7.5×R_505_) + 1
NRER	Nir-re-red normalized difference vegetation index	NIR, RE, VIS	(R_850_-R_695_)/(R_695_ + R_660_)
LCI	Leaf chlorophyll index	NIR, RE, VIS	(R_850_-R_710_)/(R_850_ + R_680_)
MTCIvar	Meris terrestrial chlorophyll index	NIR, RE	(R_850_-R_680_)/(R_680_ + R_660_)
NRI	Nitrogen reflectance index	NIR, RE	(R_555_-R_550_)/(R_555_ + R_550_)
PRI	Photochemical reflectance index	NIR, RE	(R_570_-R_530_)/(R_570_ + R_530_)
SPVI	Spectral polygon vegetation index	NIR, RE, VIS	0.4× [3.7× (R_800_-R_670_) –1.2 (R_530_-R_670_)]
SR710	Simple ratio 710	RE	R_750_/R_710_
SR680	Simple ratio 680	RE	R_800_/R_680_
RVI	Ratio vegetation index	NIR, VIS	R_810_/R_660_
VOG1	Vogelmann index	RE	R_745_/R_720_
GM	Gitelson and Merzlyak index	RE, VIS	R_750_/R_550_
MNDm	Modified normalized difference	NIR, RE, VIS	[(R_750_-R_705_)/(R_750_ + R_705_-2× R_508_)]
NDRE2	Normalized difference nir/red edge index	NIR, RE, VIS	(R_795_-R_720_)/(R_795_ + R_720_)
MCARI2	Modified chlorophyll absorption in reflectance	NIR, RE, VIS	[(R_750_-R_705_) –0.2 (R_750_-R_550_) × (R_750_/R_705_)]
TVI	Triangular vegetation index	NIR, RE, VIS	0.5× [120× (R_750_-R_550_) –200(R_670_-R_550_)]
EVI2	Enhanced vegetation indexrep	NIR, RE, VIS	2.5× (R_800_-R_670_)/(R_800_ + 6×R_670_-7.5×R_508_) + 1
REP	Red Edge position index	NIR, RE, VIS	700 + (45×R_670_ + R_778_)/2- (R_850_)/(R_735_ –R_695_)
maxR	1st Derivative Max RED index	dHVI-VIS	Max [D_660_, D_680_]
sumR	1st Derivative Sum RED index	dHVI-VIS	Σ [D_660_, D_680_]
maxRE	1st Derivative Max RE index	dHVI-RE	Max [D_690_, D_700_]
sumRE	1st Derivative Sum RE index	dHVI-RE	Σ [D_690_, D_700_]
maxLARE	1st Derivative Max LARE index	dHVI-RE	Max [D_690_, D_710_]
sumLARE	1st Derivative Sum LARE index	dHVI-RE	Σ [D_690_, D_710_]
maxNIR	1st Derivative Max NIR index	dHVI-NIR	Max [D_790_, D_840_]
sumNIR	1st Derivative Sum NIR index	dHVI-NIR	Σ [D_790_, D_840_]

Spectral bands were imported into Matlab and, for the productive, qualitative and vegetative parameters, one dataset was obtained with size {60 × 50}, composed by the average values corresponding to the 50 wavelengths, for each one of the 60 vine samples. As regards ecophysiological parameters, Ψ_pd_ and Ψ_md_ were determined on 30 vines out of the 60 plants, therefore the size of obtained dataset was equal to {30 × 50}. Partial Least Square regression (PLSr) (Naes et al., 2002) was used to build multivariate calibration models in order to evaluate the correlation performance between the biophysical parameters and hyperspectral imagery using the whole spectral range. The calibration models were calculated on the mean centered dataset and the statistical parameters used to evaluate the PLS performance were the Root Mean Square Error (RMSE) and coefficient of determination (*R*^2^); both parameters were calculated in calibration (RMSEC, *R*^2^ Cal) and in cross-validation (RMSECV, *R*^2^ CV). The optimal number of Latent Variables (LVs) was chosen by minimizing the value of RMSECV. In particular, a random cross-validation method was used, subdividing the samples in 3 deletion groups. In order to evaluate the possibility of reducing the number of wavelengths and selecting the more relevant variables for each parameter, interval-PLS (iPLS) was tested as algorithm for automatically variable selection (Norgaard et al., 2000). Briefly, iPLS consists of subdividing the whole signal into a certain number of intervals of equal length which is defined by the user. Calibration models are calculated by iteratively adding or removing intervals, according to whether the forward or reverse search strategy is considered. The most useful intervals for model calibration are identified by minimizing the RMSECV value (Orlandi et al., 2018). In this work forward iPLS was applied considering two different interval sizes: 10 and 5 variables. PLS and iPLS calibration models were elaborated and cross-validated by means of PLS-Toolbox ver. 8.9.1 (Eigenvector Research Inc., Manson, WA, United States).

## Results

### Ground Measurements

Data reported in Table 2 identify a significant within-field variability at both physiological and agronomical level. With a CV of 24%, Ψ_pd_ at veraison varied between –0.27 and –0.73 MPa suggesting a transition from slight to severe water shortage in the soil. Ψ_md_ showed lower variability (CV = 9.8%) although the calculated mean value (–1.34 MPa) highlights a likely status of relatively severe water stress. Data describing canopy growth and vine capacity depicted a highly variable vineyard condition. Indeed, total leaf area per vine varied between 0.65 and 3.43 m^2^ with a coefficient of variation of about 34% that peaked up to 78% in the case of lateral leaf area. This variability was confirmed in winter with pruning weight of 1-year canes varying between 0.16 and 1.07 kg/vine (CV = 49%), identifying the coexistence of very low and high vigor vines within the 15-row plot. Yield in high vigor vines was 11-fold higher as compared to low cropping vines as a result of bigger clusters (386 vs. 54 g) and berries (2.8 vs. 1.3 g). At harvest, a large variability in fruit composition was described for TSS (CV = 11.4%), titratable acidity (CV = 15.8%) and the variables describing phenolic composition. The highest coefficients of variation were described for malate (56.8%), anthocyanins (38.3%), and total phenolics (26%) concentration.

**TABLE 2:** Mean, minimum and maximum values, and coefficient of variation (CV%) for leaf water status, canopy growth, yield components, and fruit composition of Barbera grapevines recorded in 2020.

Variable	Mean	Min	Max	CV (%)
Ψ_pd_ (MPa)	–0.5	–0.27	–0.73	24.2
Ψ_md_ (MPa)	–1.34	–1.08	–1.64	9.8
Total leaf area (m^2^/vine)	1.85	0.65	3.43	33.8
Lateral leaf area (m^2^/vine)	0.17	0.00	0.53	77.6
Pruning weight (kg/vine)	0.48	0.16	1.07	48.7
Yield (kg/vine)	3.37	0.76	8.46	52.2
Cluster weight (g)	178.4	54.3	386.1	41.6
Berry weight (g)	2.0	1.3	2.8	20.2
TSS (°Brix)	24.4	19.0	29.3	11.4
Titratable acidity (g/L)	9.66	6.58	14.41	15.8
Malate (g/L)	2.13	0.75	5.99	56.8
Total anthocyanins (mg/g)	0.75	0.18	1.43	38.3
Total phenolics (mg/g)	1.78	0.88	2.71	26.0

### Relationship Between Narrowband HVIs and Grapevine Performances

The coefficients of determination (*R*^2^) for the linear regressions between narrowband HVIs and ground measurements are reported in Table 3.

**TABLE 3:** Coefficients of determination (*R*^2^) for linear regressions between narrowband HVIs and ground measurements.

HVIs	Ψ_pd_	Ψ_md_	Yield	Cwt	Bwt	TSS	TA	Malate	Anth	Phenols	TLA	LLA	Pwt
NDVI1	0.6***	0.23**	0.3***	0.34***	0.47***	0.2***	0.29***	0.57***	0.36***	0.43***	0.22***	0.27***	0.23***
NDVI2	0.6***	0.25**	0.28***	0.32***	0.48***	0.19***	0.3***	0.58***	0.37***	0.42***	0.22***	0.27***	0.19***
GNDVI1	0.53***	0.12	0.36***	0.38***	0.41***	0.13**	0.24***	0.48***	0.25***	0.32***	0.17***	0.23***	0.23***
RENDVI	0.52***	0.12	0.29***	0.31***	0.41***	0.12**	0.21***	0.47***	0.28***	0.34***	0.15**	0.25***	0.22***
**MTCIvar**	0.58***	0.2*	0.32***	0.35***	0.48***	0.18***	0.29***	0.58***	0.36***	0.43***	0.18***	0.26***	0.23***
SAVI	0.6***	0.25**	0.26***	0.29***	0.43***	0.21***	0.3***	0.56***	0.36***	0.39***	0.27***	0.3***	0.16**
TCARI	0.35***	0.35***	0.11*	0.14**	0.26***	0.26***	0.32***	0.44***	0.32***	0.35***	0.23***	0.2***	0.11**
MTVI	0.6***	0.28**	0.23***	0.26***	0.41***	0.22***	0.32***	0.58***	0.38***	0.4***	0.27***	0.31***	0.16**
**EVIm**	0.62***	0.28**	0.24***	0.27***	0.41***	0.25***	0.34***	0.61***	0.4***	0.45***	0.24***	0.33***	0.23***
GNDVI2	0.57***	0.23**	0.26***	0.3***	0.32***	0.21***	0.15**	0.41***	0.31***	0.37***	0.16**	0.29***	0.29***
NDRE	0.27**	0.12	0.08*	0.07*	0.07*	0.11**	0.04	0.11**	0.06	0.07*	0.02	0.13**	0.11*
LCI	0.49***	0.09	0.28***	0.28***	0.28***	0.08*	0.21***	0.39***	0.2***	0.25***	0.09*	0.18***	0.21***
MTVI2	0.63***	0.29**	0.23***	0.25***	0.4***	0.22***	0.32***	0.59***	0.38***	0.41***	0.27***	0.31***	0.17**
**NDVI3**	0.65***	0.26**	0.25***	0.29***	0.44***	0.21***	0.27***	0.58***	0.38***	0.44***	0.23***	0.32***	0.24***
NRI	0.19*	0.04	0.15**	0.17**	0.26***	0.05	0.13**	0.23***	0.09*	0.12**	0.1*	0.04	0.03
PRI	0.13	0.13	0.01	0.01	0.02	0.08*	0.07*	0.15**	0.11**	0.14**	0.01	0.05	0.02
SPVI	0.63***	0.28**	0.23***	0.26***	0.4***	0.21***	0.31***	0.58***	0.37***	0.39***	0.27***	0.32***	0.17**
SR710	0.39***	0.13	0.21***	0.21***	0.21***	0.09*	0.15**	0.29***	0.19***	0.22***	0.07*	0.16**	0.12**
SR680	0.63***	0.24**	0.3***	0.31***	0.47***	0.19***	0.3***	0.62***	0.39***	0.43***	0.22***	0.25***	0.19***
**RVI**	0.63***	0.36***	0.3***	0.33***	0.48***	0.25***	0.36***	0.63***	0.42***	0.48***	0.21***	0.28***	0.23***
VOG1	0.12	0.03	0.13**	0.08*	0.06	0.09*	0.02	0.08*	0.11*	0.13**	0	0.05	0.17**
GM	0.53***	0.22*	0.28***	0.32***	0.36***	0.19***	0.23***	0.46***	0.3***	0.35***	0.18***	0.28***	0.21***
MNDm	0.23**	0.06	0.12**	0.11*	0.13**	0.12**	0.08*	0.19***	0.14**	0.19***	0.01	0.14**	0.16**
NDRE2	0.3**	0.06	0.19***	0.19***	0.2***	0.08*	0.06	0.15**	0.15**	0.16**	0.04	0.08*	0.13**
MCARI2	0.47***	0.19*	0.1*	0.13**	0.23***	0.24***	0.23***	0.43***	0.3***	0.34***	0.2***	0.31***	0.16**
TVI	0.61***	0.31**	0.19***	0.22***	0.37***	0.26***	0.31***	0.58***	0.39***	0.44***	0.25***	0.33***	0.19***
EVI2	0.63***	0.3**	0.23***	0.25***	0.39***	0.22***	0.31***	0.57***	0.37***	0.4***	0.26***	0.33***	0.17**
REP	0.4***	0.1	0.14**	0.17**	0.28***	0.13**	0.08*	0.3***	0.19***	0.24***	0.18***	0.24***	0.12**
maxR	0.08	0.09	0	0	0.03	0.1*	0.11**	0.08*	0.04	0.04	0.03	0.09*	0.01
sumR	0	0.05	0.04	0.05	0.07*	0.08*	0.1*	0.07	0.05	0.04	0.08*	0.06	0
maxRE	0.49***	0.23**	0.15**	0.19***	0.37***	0.24***	0.24***	0.52***	0.37***	0.41***	0.26***	0.3***	0.16**
sumRE	0.52***	0.28**	0.14**	0.18***	0.36***	0.21***	0.23***	0.53***	0.36***	0.4***	0.3***	0.28***	0.15**
**maxLARE**	0.59***	0.26**	0.19***	0.2***	0.34***	0.31***	0.34***	0.56***	0.4***	0.43***	0.24***	0.34***	0.2***
sumLARE	0.59***	0.28**	0.16**	0.2***	0.36***	0.25***	0.29***	0.57***	0.38***	0.43***	0.27***	0.32***	0.19***
maxNIR	0.05	0	0.03	0.04	0	0	0.01	0.01	0	0	0.02	0.04	0.01
sumNIR	0	0.04	0.01	0.01	0.01	0.03	0.03	0.01	0	0.01	0.01	0	0.02

*Within each column the highest R^2^ values are highlighted.*

***, **, * and () indicate p < 0.0001, < 0.001, < 0.01, and > 0.01, respectively.

*Narrowband HVIs reported in the first column are described in Table 1. Ψ_pd_, pre-dawn leaf water potential; Ψ_md_, mid-day leaf water potential; Cwt, cluster weight; Bwt, berry weight; TSS, total soluble solids; TA, titratable acidity; Anth, total anthocyanins; TLA, total leaf area; LLA, lateral leaf area; Pwt, pruning weight.*

Generally, the more performing narrowband indices were RVI, EVIm, maxLARE, MTCIvar, and NDVI3. Conversely, poor correlations were achieved between ground measurements and maxNIR, sumNIR, sumR, maxR, NRI, and PRI. As regards the ecophysiological parameters, the majority of narrowband indices showed a good correlation with Ψ_pd_. In particular, the closest correlations were obtained with NDVI3 (*R*^2^ = 0.65), RVI (*R*^2^ = 0.63), and SR680 (*R*^2^ = 0.63). Conversely, weaker relationships were found between Ψ_md_ and the narrowband indices; however, the RVI was confirmed to be the most efficient index (*R*^2^ = 0.36). For yield components, best correlations were achieved between berry weight (Bwt) and NDVI2, MTCIva, RVI sharing an *R*^2^ of 0.48. Slightly worse correlations were found for yield and cluster weight (Cwt); for both parameters the more performing indices were NDVI1, GNDVI and MTCIvar with an *R*^2^ from 0.35 to 0.30. Among the qualitative parameters, satisfactory correlations were achieved between malate and the majority of narrowband indices. In particular, the most fitting was RVI (*R*^2^ = 0.63); however, equivalent results were also achieved using SR680 (*R*^2^ = 0.62) and EVIm (*R*^2^ = 0.61). Conversely, the same narrowband indices showed weaker correlations with titratable acidity (*R*^2^ from 0.36 to 0.30). Furthermore, quite poor relationships were found between TSS and the narrowband indices. In regard to total anthocyanins and phenols, the most performing indices were RVI and EVIm with an *R*^2^ from 0.48 to 0.40, respectively. Compared with the other ground measurements, the worst performances were obtained for vegetative parameters. Correlation between lateral leaf area (LLA) and maxLARE yielded an *R*^2^ of 0.34, whereas total leaf area (TLA) and pruning weight (Pwt) per vine achieved an *R*^2^ equal to 0.30 and 0.29 when regressed over sumRE and GNDVI2, respectively.

### Relationship Between Partial Least Square Models and Grapevine Performances

**FIGURE 4:**
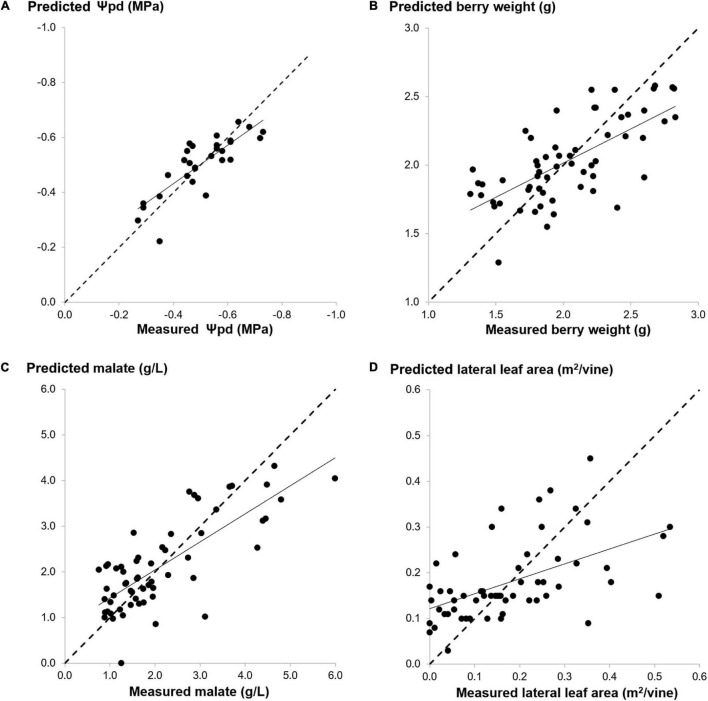
Predicted values vs. ground-based measurements of the best calibration performances of multivariate models achieved for each biophysical parameter group: Ψ_pd_ for ecophysiological Y [**(A)**, *R*^2^ CV = 0.65, RMSECV = 0.07 MPa], Bwt for productive Y [**(B)**, *R*^2^ CV = 0.46, RMSECV = 0.30 g], Malate for qualitative Y [**(C)**, *R*^2^ CV = 0.59, RMSECV = 0.78 g/L], LLA for vegetative Y [**(D)**, *R*^2^ CV = 0.31, RMSECV = 0.11 m2/vine].The dotted line indicates a regression with slope = 1.

For each Y variable, the results of the calibration performance are reported in Table 4. Overall, the best correlation performances were obtained for the parameters identifying fruit composition and, among them, malic acid (*R*^2^ CV = 0.59), total phenols (*R*^2^ CV = 0.41), and total anthocyanins (*R*^2^ CV = 0.36). Conversely, the worse correlations were obtained for the vegetative parameters and, among them, pruning weight (*R*^2^ CV = 0.27), and total leaf area (*R*^2^ CV = 0.06). The overall best calibration performance was shown by ψ_pd_ (*R*^2^ CV = 0.65-RMSECV = 0.07 MPa). This model was built using only 15 variables (three intervals made of 5 variables) out of 50 original bands. The selected regions include wavelengths belonging to 549–663 nm and 761–794 nm. The measured vs. the predicted values of ψ_pd_ are reported in Figure 4A. Conversely, it was impossible to obtain a good correlation for ψ_md_ (*R*^2^ CV = 0.22-RMSECV = 0.10 MPa).

**TABLE 4:** Results of PLS and iPLS models.

Y	Calibration method	iPLS interval size	Selected Bands (nm)	LVs	RMSEC	RMSECV	*R*^2^ cal	*R*^2^ CV
Ψ_pd_	PLS	–	–	1	0.07	0.08	0.65	0.61
	iPLS	10	590:704	3	0.06	0.08	0.76	0.61
	**iPLS**	**5**	**549:663 761:794**	**3**	**0.06**	**0.08**	**0.77**	**0.65**
Ψ_md_	**PLS**	–	–	**1**	**0.10**	**0.10**	**0.32**	**0.27**
	iPLS	10	590:794	1	0.10	0.11	0.33	0.18
	iPLS	5	802:835	2	0.09	0.10	0.38	0.22
Yield	PLS	–	–	3	1.25	1.46	0.42	0.21
	iPLS	10	508:704	2	1.34	1.44	0.32	0.23
	**iPLS**	**5**	**508:541 590:663 712:753**	**2**	**1.30**	**1.40**	**0.37**	**0.26**
Cwt	PLS	–	–	3	0.06	0.07	0.39	0.24
	**iPLS**	**10**	**508:704**	**2**	**0.06**	**0.06**	**0.35**	**0.27**
	iPLS	5	508:541 671:704	3	0.06	0.07	0.39	0.23
Bwt	PLS	–	–	3	0.28	0.32	0.53	0.38
	**iPLS**	**10**	**590:704**	**2**	**0.28**	**0.30**	**0.53**	**0.46**
	iPLS	5	549:753	3	0.27	0.31	0.56	0.44
TSS	PLS	–	–	1	2.37	2.47	0.27	0.21
	iPLS	10	590:794	1	2.35	2.41	0.28	0.24
	**iPLS**	**5**	**549:582 671:794**	**1**	**2.35**	**2.41**	**0.28**	**0.24**
TA	PLS	–	–	1	1.27	1.36	0.31	0.21
	iPLS	10	508:794	1	1.29	1.35	0.29	0.21
	**iPLS**	**5**	**508:541 802:835**	**1**	**1.27**	**1.32**	**0.31**	**0.25**
Malate	PLS	–	–	1	0.78	0.85	0.57	0.51
	iPLS	10	508:704	2	0.73	0.83	0.64	0.53
	**iPLS**	**5**	**590:704**	**2**	**0.74**	**0.78**	**0.63**	**0.59**
Anth	PLS	–	–	1	0.23	0.23	0.39	0.36
	iPLS	10	712:794	1	0.23	0.24	0.39	0.32
	**iPLS**	**5**	**549:582 712:753**	**1**	**0.23**	**0.23**	**0.38**	**0.36**
Phenols	PLS	–	–	1	0.35	0.36	0.43	0.40
	iPLS	10	712:794	1	0.35	0.36	0.44	0.41
	**iPLS**	**5**	**16:20 26:30**	**3**	**0.33**	**0.36**	**0.50**	**0.41**
TLA	PLS	–	–	1	0.55	0.56	0.07	0.04
	**iPLS**	**10**	**590:704**	**2**	**0.52**	**0.55**	**0.16**	**0.06**
	iPLS	5	671:704	1	0.52	0.56	0.15	0.05
LLA	PLS	–	–	1	0.11	0.11	0.35	0.27
	iPLS	10	712:794	2	0.11	0.11	0.37	0.29
	**iPLS**	**5**	**712:753**	**1**	**0.11**	**0.11**	**0.35**	**0.31**
Pwt	**PLS**	–	–	**2**	**0.18**	**0.20**	**0.38**	**0.27**
	iPLS	10	712:794	2	0.19	0.20	0.36	0.27
	iPLS	5	761:794	2	0.19	0.20	0.35	0.25

*Within each Y parameter the best calibration performance is reported in bold.*

Concerning the yield and its components (Cwt and Bwt), a satisfactory correlation was obtained for berry weight (R^2^ CV = 0.46-RMSECV = 0.30 g). This model was built by means of iPLS using one interval made of 10 bands with the selected wavelengths belonging to 590–704 nm (Figure 4B). Among the vegetative parameters, the best correlation performance was achieved for LLA (*R*^2^ CV = 0.31-RMSECV = 0.11 m^2^/vine) by means of iPLS. This result was obtained considering only the wavelengths belonging to 712–753 nm, that were selected using an interval width of 5 variables (Figure 4D). When compared with the corresponding PLS model calculated on the whole spectral range, the iPLS model generally resulted in equal values or a slight reduction of the RMSECV value. However, the variable selection also led to reducing the number of wavelengths while selecting the more relevant variables for each Y parameter. The best improvement was achieved for malic acid: the RMSECV value obtained with iPLS is equal to 0.78 g/L instead of an RMSECV value equal to 0.85 g/L obtained with PLS (Figure 4C). The variable selection allowed a model to be built using only 10 bands (two intervals made of 5 variables) out of 50 original bands. The selected wavelengths belonging to 590–704 nm.

## Discussion

Interpretation of the *R*^2^ data reported in Table 3 vs. means, range of variation and coefficient of variation (CV) of agronomic and physiological variables is quite puzzling. In general terms, for a given index, closer correlation is expected any time a given variable, primarily due to soil heterogeneity, shows a larger degree of variability (Trought et al., 2008; Baluja et al., 2013; Squeri et al., 2019; Gatti et al., 2021). This concept seems to hold, for instance, when RVI, NDVI3, SR680, and EVI2 are correlated with Ψ_pd_ and Ψ_md_. In all cases *R*^2^ calculated for Ψ_pd_ is more or less halved when referred to Ψ_md._ The most obvious reason for such a drop seems to be the lower CV (9.8%) calculated for Ψ_md_ which testifies to a fairly narrow range of variation. As a matter of fact, there is no reason to think that differential sensitivity of the two types of water potential are due to different sampling methodology (both were assessed through the pressure chamber method). This outcome is not encouraging if HS indices are expected to be used as a replacement for the tedious pressure chamber method for total midday or stem water potential measurements; this is not just because *R*^2^ are rather low, but also because given the recorded mean Ψ_md_ (–1.34 MPa) and calculated RMSECV of about –0.1 MPa the same mean values can represent a condition of either moderate of severe stress depending upon the concurrent evaporative demand (VPD on DOY 213 was 4 kPa). Vice versa, Pearson correlation values found for Ψ_pd_ vs. NDVI3, SR680 and RVI reveal chances that a quite reliable, yet otherwise slow and laborious reading such as pre-dawn water potential, could be replaced with a fast, non-destructive UAV-hyperspectral protocol resulting in a very high resolution mapping of soil and plant water status. However, when the same concept is applied to yield including two of its main components, the hypothesis basically fails. Results concerning yield per vine and two of its main components (i.e., berry and cluster weight) showed that berry weight was slightly more responsive to some indices such as NDVI2, MTCIvar, and RVI (*R*^2^ = 0.48) although the other components (yield and Cwt) did show higher CVs than berry weight. The hypothesis is that total yield is largely affected by cultural and endogenous factors (e.g., varietal fruitfulness, bud induction, bud load, summer pruning operations) whose description through a spectral signature is more troublesome. For instance, floral bud induction for next season cropping is typically decided in grapevine the season before the image is taken and it is controlled, among several factors, by specific growth and environmental conditions at that time (May, 2004). As per final grape composition, the overall mild correlations found for all VIs vs. TSS (°Brix) at harvest support recent work by Suarez et al. (2021), who also reported non-significant correlations for data taken on Shiraz. It is quite notable from our study that a few VIs which already provided good correlations with Ψ_pd_ (EVIm, SR680, RVI), were those also having close correlation with malic acid concentration at harvest (*R*^2^ = 0.61–0.63) as well as with total anthocyanins and phenols concentration (*R*^2^ = 0.40–0.48). This response is quite valuable for at least two main reasons: (i) it is indeed not a case that malate, color and phenolics are well known to be quite responsive to local canopy microclimate conditions and namely those pertaining to the fruit zone. Such high correlations demonstrate that the proposed indices do have the potential to predict changes in fruit composition at harvest especially for the parameters that are highly dependent on light and thermal conditions around the cluster which have proven to be the main drivers for either synthesis or degradation of the above components (Downey et al., 2004; Pereira et al., 2006; Mori et al., 2007; Sweetman et al., 2009). Such changes might be a function of the inherent vine vigor, type of training system, timing and extent of leaf removal or shoot thinning (Poni et al., 2018); (ii) taking RVI as the best example, it is viticulturally quite relevant and useful to have HS indices warranting good correlation with a range of variables representing plant water status (e.g., Ψ_pd_), crop potential (e.g., fresh berry weight), and degree of maturity (e.g., TSS, malate, anthocyanins, and phenols). We also feel that this is the first time this achievement is reported, as in the Suarez et al. (2021) paper the very close correlation that several HS indices show with a few terpene compounds is not reflected in any significant correlation with either color and phenolics.

Many approaches have been proposed to evaluate the biophysical parameters of different plants by means of hyperspectral data (Ismail and Mutanga, 2010; Doktor et al., 2014; Yao et al., 2015). Among them, Atzbergera et al. (2010) demonstrated that PLS is better performing then other methods in order to extract the information by the whole spectral range for evaluation of the canopy chlorophyll content in winter wheat. In addition, the current study investigates the possibility of selecting the most relevant feature by means of iPLS. In particular, the results proved that the variable selection allowed the RMSECV values to be slightly reduced or to obtain equal values compared to the results obtained using the whole spectral range.

Pôças et al. (2017) developed an effective approach based on hyperspectral reflectance data aimed at monitoring the grapevine water status. However, the results obtained in this study demonstrated the possibility of assessing other biophysical parameters, such as productive ones. In particular, a good performance was obtained for Bwt with a RMSECV value equal to 0.30 g, using only 10 wavelengths belonging to 590–704 nm.

Furthermore, the results of this study showed better performance than those obtained by Suarez et al. (2021) when using plant traits derived from physical model inversion of hyperspectral imagery for the evaluation of qualitative parameters of grapevine, such as phenolic content. Considering that the grapevine is a complex system characterized by a dynamic balance between vegetative and productive features, another strong point of this work is proving the potential of a hyperspectral imaging sensor on the main key factors of the “vine-ecosystem.” In fact, compared to other cited works, a wide scenario has been explored, both functional aspects related to the eco-physiological state, as well as the vegetative growth and finally the quantitative and qualitative productive response at the end of the cycle. To understand the real effectiveness of a non-destructive optical techniques it is in fact necessary to have a vision of the main traits of the “vine-ecosystem,” not just focusing on single or few aspects. Regarding the high cost of hyperspectral imaging technology, there are very few works in the literature using UAV equipped with these cameras in field conditions, especially with the wide ground truth dataset collected here. Furthermore, another limitation of the research on this topic is the high level of experience necessary to identify and apply correct in-flight data acquisition and management protocols, especially given the lack of ready-to-use software to perform the complete processing workflow of the hypercubes.

Due to their inherent structure, vineyards pose a specific challenge for remote sensing analysis (Singh et al., 2022). This is due not just to a quite typical discontinuous canopy cover which introduces the issue of “mixels” handling, rather to at least three other peculiar features: (i) vines are extremely sensitive to any factors causing spatial and temporal variation in growth and yield and, on top of them, soil heterogeneity; (ii) large variability in training systems (i.e., vigor, geometry, distance between rows) originates complex interactions in terms of background and shade, including also large diurnal variation, and (iii) more than in any other orchard system, interference exerted by the presence of portions of bare or grassed soil can be of utmost complexity. All of this justifies why remote sensing images of vineyards must be processed to separate canopy pixels from the background. Moreover, considering that viticulture is one of the most profitable agriculture sectors, digital agriculture solutions play a key role in the decision-making processes for grape production respect to other lower valuable crops. Viticulture is a key socioeconomic and cultural sector in many countries and regions worldwide, with a high economic impact in the network of all relevant industry branches of the supply and distribution chains. The latest report of the International Organization of Vine and Wine (OIV, 2019), it is estimated that the world vineyards cover an area of approximately 7.449 million ha (2018). Concerning the winemaking sector, global wine production was 292 million hl in 2018, and wine trade in monetary value has been growing continuously to reach a record-breaking value of approximately EUR 30,000 million in 2018. For these suggestions, however, studies of this type are necessary to guide the technology transfer on solutions that have been adequately tested (Tardaguila et al., 2021; Di Gennaro et al., 2022). Another key issue is the challenge of climate change and the need to describe plant processes at a very detailed level, using a large number of inputs, may currently preclude the applicability of simulation models as decision support tools for farmers. In fact, models coupled with the use of new technologies such as UAV and hyperspectral imagery may represent the most appropriate management practices in the future.

The main limitation in this work is due to the fact that is more reasonable to continuously measure the spectrum and use it to estimate the dynamic changes of various attributes, and finally analyze the yield and quality, but in our case a single flight was used, identified as the best acquisition date in line with our previous studies on vineyard (Matese and Di Gennaro, 2021), to characterize the physiological and biochemical parameters at harvest. Moreover, using this approach the aim was to develop a more prompt predictive model for farmer and thus an operational tool for characterizing quanti-qualitative parameters in the vineyard.

## Conclusion

On the agronomic side, the calculation of indices derived from HS data cubes has shown very promising potential for: (i) achieving high correlations with variables that are more closely linked to local canopy microclimate conditions, such as malic acid, total anthocyanins and phenols concentration and (ii) identifying specific indices with the ability to concurrently describe several vine traits including water status, cropping potential and ripening patterns. The novelty of this work is represented by the first assessment of a hyperspectral UAV dataset with grapevine parameters using several hyperspectral narrowband indices and multivariate PLS regressions. The strength of this research is the study of hyperspectral data acquired by UAV in field conditions by examining the expression of the entire vine ecosystem, from the physiological state, to descriptors of vine vegetative development, and finally on grapes production and quality. The results obtained by applying a wide spectrum of VIs allow alternative solutions to the traditional and time-consuming ground measurements to be identified, which provide the best accuracy, but frequently lead to a limitation for representative sampling in a large vineyard. Above all for the monitoring of physiological parameters, which must be done in a short time as they are extremely influenced by the variability of environmental conditions during the day, such as air temperature and humidity or the intensity and angle of solar radiation. A correct non-destructive estimation of key parameters in the vineyard represents a powerful tool to support the winegrower in optimal vineyard management, both for agronomic input choices and planning the best harvest date. Further work is needed to explore the robustness of this methodology on different phenological stages of grapevines and on the use of innovative Machine Learning algorithms.

## Data Availability Statement

The datasets presented in this article are not readily available because data are available from the authors upon reasonable request and with permission of the company that hosted the study. Requests to access the datasets should be directed to SD, salvatorefilippo.digennaro@cnr.it.

## Author Contributions

AM and SP designed the experiment and coordinated the activity. SD, AM, GO, and MG performed the data acquisition and data processing. SD, AM, MG, and SP wrote and reviewed the manuscript. All authors read and approved the final manuscript.

## Conflict of Interest

The authors declare that the research was conducted in the absence of any commercial or financial relationships that could be construed as a potential conflict of interest.

## Publisher’s Note

All claims expressed in this article are solely those of the authors and do not necessarily represent those of their affiliated organizations, or those of the publisher, the editors and the reviewers. Any product that may be evaluated in this article, or claim that may be made by its manufacturer, is not guaranteed or endorsed by the publisher.

## Footnotes

1
QGIS.org

2
http://www.qgis.org
